# Comparative Genomic Analysis of* Delftia tsuruhatensis* MTQ3 and the Identification of Functional NRPS Genes for Siderophore Production

**DOI:** 10.1155/2016/3687619

**Published:** 2016-10-25

**Authors:** Haimeng Guo, Yanan Yang, Kai Liu, Wenfeng Xu, Jianyong Gao, Hairong Duan, Binghai Du, Yanqin Ding, Chengqiang Wang

**Affiliations:** ^1^College of Life Sciences/Shandong Key Laboratory of Agricultural Microbiology, Shandong Agricultural University, Tai'an, China; ^2^State Key Laboratory of Nutrition Resources Integrated Utilization, Linyi, China; ^3^GENEWIZ, Suzhou, China

## Abstract

Plant growth-promoting rhizobacteria (PGPR) are a group of rhizosphere bacteria that promote plant growth.* Delftia tsuruhatensis* MTQ3 is a member of PGPR that produces siderophores. The draft genome sequence of MTQ3 has been reported. Here, we analyzed the genome sequence of MTQ3 and performed a comparative genome analysis of four sequenced* Delftia* strains, revealing genetic relationships among these strains. In addition, genes responsible for bacteriocin and nonribosomal peptide synthesis were detected in the genomes of each strain. To reveal the functions of NRPS genes in siderophore production in* D. tsuruhatensis* MTQ3, three NRPS genes were knocked out to obtain the three mutants MTQ3-Δ1941, MTQ3-Δ1945, and MTQ3-Δ1946, which were compared with the wild-type strain. In qualitative and quantitative analyses using CAS assay, the mutants failed to produce siderophores. Accordingly, the NRPS genes in MTQ3 were functionally related to siderophore production. These results clarify one mechanism by which plant growth is promoted in MTQ3 and have important applications in agricultural production.

## 1. Introduction

Plant growth-promoting rhizobacteria (PGPR) could promote plant growth by a variety of mechanisms, such as siderophore production [1], antibiotics secretion, phytohormone generation, and the induction of systemic resistance [2, 3]. PGPR have an important value in agricultural production.

Iron is essential to the growth of plants, but soluble iron is often insufficient in soil. Siderophores, which are low-molecular-weight molecules with a high affinity for ferric iron [4] secreted by PGPR, can mitigate that limitation, to some extent. Many microorganisms, including bacteria and fungi, can secrete siderophores [5]. In general, siderophores can be separated into two types based on their biosynthetic mechanism. One type depends on the nonribosomal peptide synthetase (NRPS) pathway, and the other is NRPS-independent [6].

*Delftia* has the ability to biodegrade organic pollutants, such as aniline [7], phenolic compounds [8], 2,4-dichlorophenoxyacetic acid (2,4-d) [9], and acetochlor.* Delftia tsuruhatensis* MTQ3 (MTQ3 for short) has been isolated by our group from the rhizosphere of tobacco in Guizhou, China. As an environmentally friendly PGPR, MTQ3 exhibits the potential to produce siderophores. Although species in the genus* Delftia* have been described as PGPR [10], their ability to produce siderophores has not been verified. The draft genome sequence of MTQ3 was formerly reported [11], but the mechanism of siderophore production is unclear.

In this work, a comparative genomic analysis of MTQ3 and the related genome sequences of* Delftia* sp. Cs1-4,* D. acidovorans* SPH-1, and* D. tsuruhatensis* 391 was performed. We characterized the genetic differences between the four* Delftia* strains. According to the genome annotation of MTQ3, a gene cluster that included three NRPS genes on scaffold 2 was found. The NRPS modular organizations were predicted using the PKS/NRPS Analysis website [15]. We constructed NRPS gene knockout mutants to analyze gene function with respect to siderophore biosynthesis.

## 2. Materials and Methods

### 2.1. Bacterial Strains and Plasmids

Bacterial strains and plasmids used in this study are presented in Table 1.* D. tsuruhatensis* MTQ3 was used as the wild-type strain to conduct mutants. Three NRPSs (AA671_12415, AA671_12425, and AA671_12430) were knocked out to generate three mutants: MTQ3-Δ1941, MTQ3-Δ1945, and MTQ3-Δ1946, respectively.* Escherichia coli* strains were used for the construction of plasmids.

### 2.2. DNA Extraction

Chromosomal DNA of MTQ3 was extracted from 1 mL of overnight culture using the TIANamp Bacteria DNA Kit (TIANGEN, Beijing, China) according to the manufacturer's instructions.

### 2.3. Primers and Gene Amplification

All primers used in this study are presented in Table 2. The target genes orf-1941 (AA671_12415), orf-1945 (AA671_12425), and orf-1946 (AA671_12430) were amplified from the genomic DNA of MTQ3 by PCR using relevant primers. PCR fragments were electrophoresed, excised, and purified using the TIANgel Midi Purification Kit (TIANGEN) [16].

### 2.4. Molecular Phylogenetic Analysis

To make phylogenetic inferences, the 16S rRNA gene was amplified from the genomic DNA using the universal bacterial 16S rRNA gene primers 27F and 1492R. PCR products were sequenced by GENEWIZ (Jiangsu, China). The 16S rRNA gene sequence was Blast-searched [17, 18] against the NCBI database [19] to identify homologous sequences from other species. A phylogenetic tree were generated using MEGA5 [20].

### 2.5. Comparative Genome Analysis

The three* Delftia* genomes* Delftia* sp. Cs1-4 (NC-015563),* D. acidovorans* SPH-1 (NC-010002), and* D. tsuruhatensis* 391 (JNWH00000000) obtained from GenBank were used for a comparative genome analysis with MTQ3 (LCZH00000000). The clusters of orthologous groups (COG) functional categories were analyzed by exploring all predicted proteins in the COG database using BLASTP [21]. Nonribosomal peptide and polyketide synthesis gene clusters were recognized using anti-SMASH (http://antismash.secondarymetabolites.org/help.html) [22], and their structures were compared to those of other known clusters.

### 2.6. Gene Knockout

All molecular genetic procedures for the genes orf-1941, orf-1945, and orf-1946 were performed according to the methods described in [23].

### 2.7. Medium and Cultivation

For plasmid construction,* E. coli* strains were cultured in Luria-Bertani (LB) medium with gentamycin (50 *μ*g/mL) or kanamycin (100 *μ*g/mL), as needed, at 37°C.* D. tsuruhatensis* MTQ3 and the mutants were grown in LB medium with rifampicin (10 *μ*g/mL). The 5% sucrose plus LB plates with kanamycin and rifampicin were used to screen the recombinant strains. A CAS-agar plate [24] was used to qualitatively detect siderophores. For the quantitative analysis of siderophores, sucrose-asparagine (SA) medium was necessary, which included (per liter) 20 g of sucrose, 2.0 g of l-asparagine, 0.5 g of K_2_HPO_4_, and 0.5 g of MgSO_4_·7H_2_O [25].

### 2.8. Qualitative and Quantitative Analyses of Siderophores

Single clones of strains MTQ3, MTQ3-Δ1941, MTQ3-Δ1945, and MTQ3-Δ1946 were cultivated in LB plates at 37°C overnight. Then, the bacterial lawn was inoculated on a CAS-agar plate for cultivation at 37°C for 2-3 days, and the development of a color ring was monitored.

Single clones of MTQ3, MTQ3-Δ1941, MTQ3-Δ1945, and MTQ3-Δ1946 were inoculated into 10 mL of sucrose-asparagine (SA) liquid medium and incubated at 180 rpm and 37°C for two days. SA liquid medium [25] contains 20 g L^−1^ sucrose, 2.0 g L^−1^ l-asparagine, 0.5 g L^−1^ K_2_HPO_4_, and 0.5 g L^−1^ MgSO_4_·7H_2_O. The supernatants of liquid cultures (*A*_*s*_) were collected by centrifugation at 10000 rpm for 15 min and then mixed with the CAS assay solution (at a volume ratio of 1 : 1) [26]. Uninoculated SA liquid medium was also treated following the same protocol as a reference (*A*_*r*_). After a 1-hour reaction period, the absorbances of *A*_*s*_ and *A*_*r*_ were detected at 630 nm [27]. Siderophore units were estimated as [(*A*_*r*_ − *A*_*s*_)/*A*_*r*_] × 100 = siderophore units (%) [24]. Units not exceeding 10 were regarded as negative and no color change was found in the mixture.

## 3. Results

### 3.1. Phylogenetic Analyses

**Figure 1:**
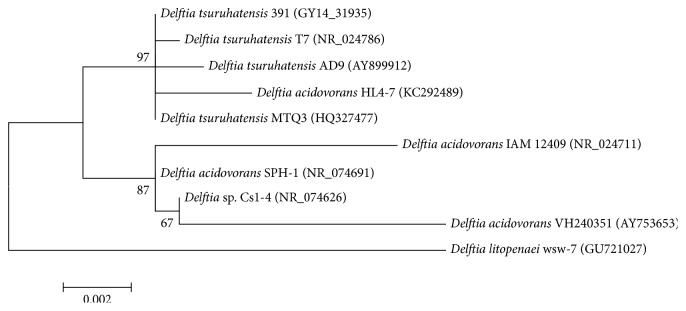
Phylogenetic tree based on the 16S rRNA gene sequence of MTQ3 and related strains. The phylogenetic tree was constructed using the maximum likelihood method with 1000 bootstrap replications. GenBank accession numbers are presented in brackets next to the species names.

A phylogenetic tree was constructed based on the 16S rRNA sequences of* Delftia* spp. [28] in MEGA5 (Figure 1). These results suggested that strain MTQ3 had high homology with* D. tsuruhatensis*.

### 3.2. Comparative Analysis with Strains Cs1-4, SPH-1, and 391

The general features of the four genomes are summarized in Table 3. There was clear variation in genome size. Specifically, the genome size varied from 5.7 Mb in MTQ3 to 6.7 Mb in SPH-1. Chromosomal coding DNA sequences (CDS) varied between 4103 in strain 391 and 6040 in SPH-1. The (G + C) mol% of the species shared a mean value of 66.60% and no strain differed from the mean value by >0.3%. These results indicated a genetic relationship between the species, to some extent.

**Figure 2:**
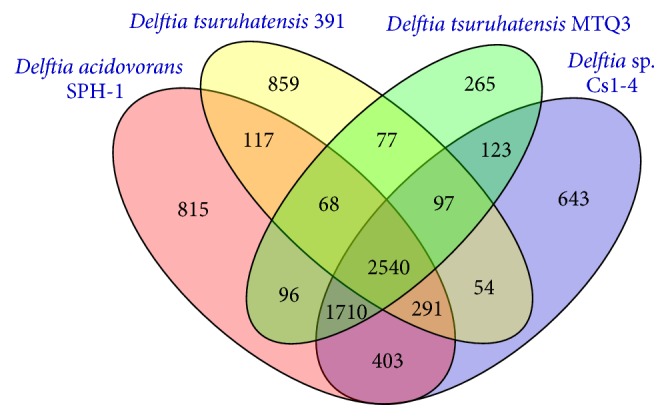
Comparison of the gene contents of MTQ3, Cs1-4, SPH-1, and strain 391.

To compare these genomes, the orthologous and unique genes among the four genomes were analyzed (Figure 2) [21]. The orthologous genes are contained in all strains, while the unique genes are owned by only one strain. A total of 2540 orthologous genes were shared and represented 51.05%, 43.34%, 42.05%, and 61.91% of all genes in MTQ3, Cs1-4, SPH-1, and 391, respectively. MTQ3 shared 4470, 4414, and 2782 orthologous genes with Cs1-4, SPH-1, and strain 391, respectively. Meanwhile, MTQ3 possessed the least unique genes and accounted for 5.14% of all genes in its genome, but strain 391 contained the most unique genes and accounted for 20.94% in its genome. That might indicate that the genes of MTQ3 presented more conserved core genome for* D. tsuruhatensis*.

**Figure 3:**
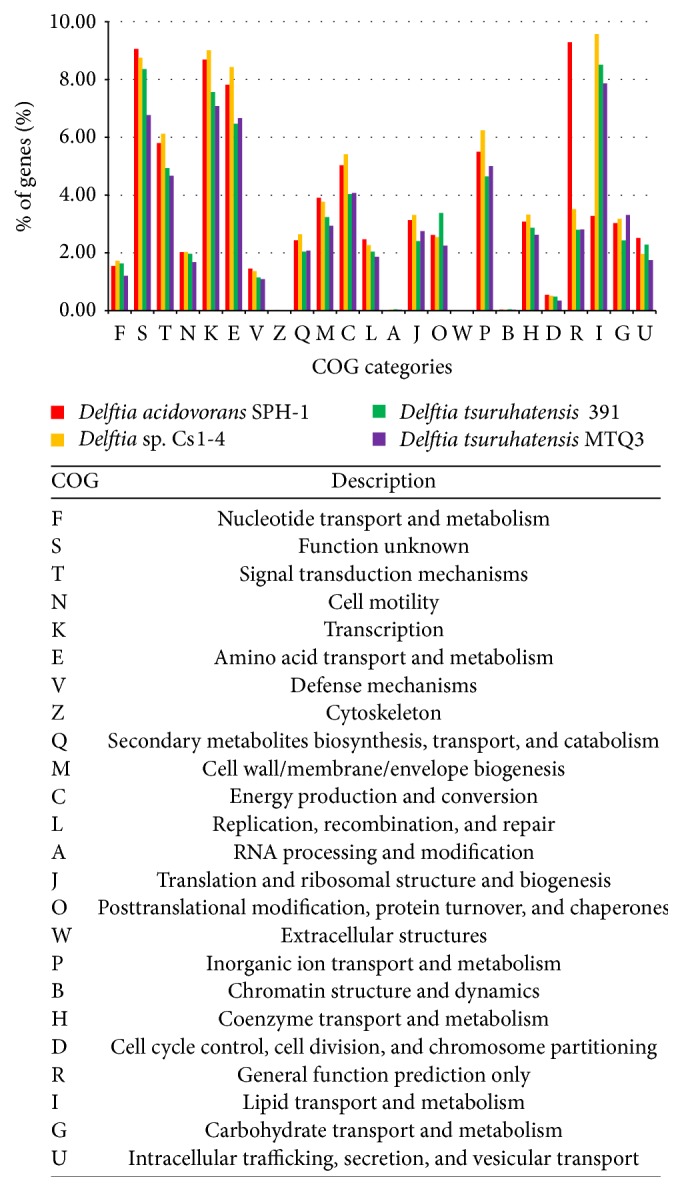
COG functional categorization of sequenced* Delftia* genomes.

Based on the COG-based analysis, the genes of these four genomes showed some similarities with respect to the distributions of COG categories (Figure 3). For the four genomes, genes related to transcription, amino acid transport and metabolism, and lipid transport and metabolism (COG categories K, E, and I, resp.) were relatively abundant functional categories, in addition to R and S, which represent general predicted functions and unknown functions, respectively, and provide little information regarding protein function [29]. The genome of MTQ3 included a larger proportion of genes involved in carbohydrate transport and metabolism compared with the other three genomes.

**Figure 4:**
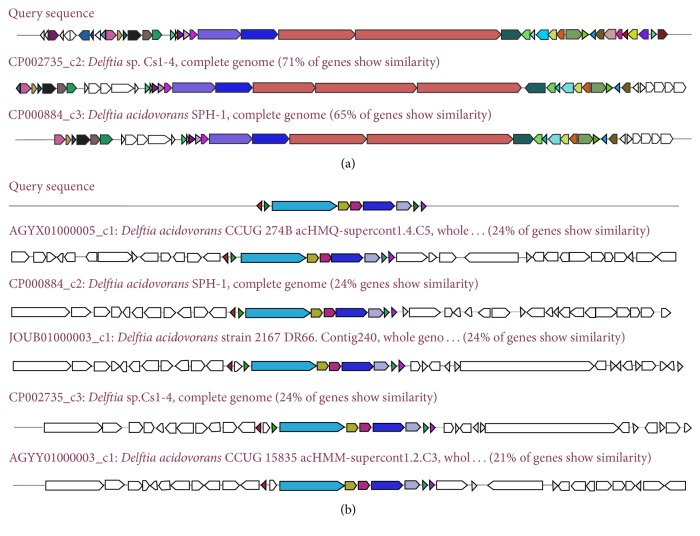
Nonribosomal peptide and polyketide synthesis clusters. (a) NRPS gene cluster. (b) Bacitracin synthesis cluster. The query sequence refers to the sequence of MTQ3 (http://antismash.secondarymetabolites.org/help.html).

Bacteriocins, which are antimicrobial peptides or proteins produced by bacteria, could enhance environmental adaption. Enzymes related to the synthesis of nonribosomal peptides (NRP) and polyketides (PK) are modular and composed of a series of domains including adenylation, thiolation, condensation, and esterification domains [29]. We compared the NRPS gene cluster between the four genomes of* Delftia*, which are summarized in Figure 4. Bacteriocin synthetic gene clusters are also listed in Figure 4(b). The query sequence refers to the sequence of MTQ3. These results showed that not all* Delftia* genomes have identical clusters of NRPS and bacteriocin synthesis genes. These differences may reflect adaptations of the strains to their specific environments.

### 3.3. The Knockout of NRPSs in MTQ3

**Figure 5:**
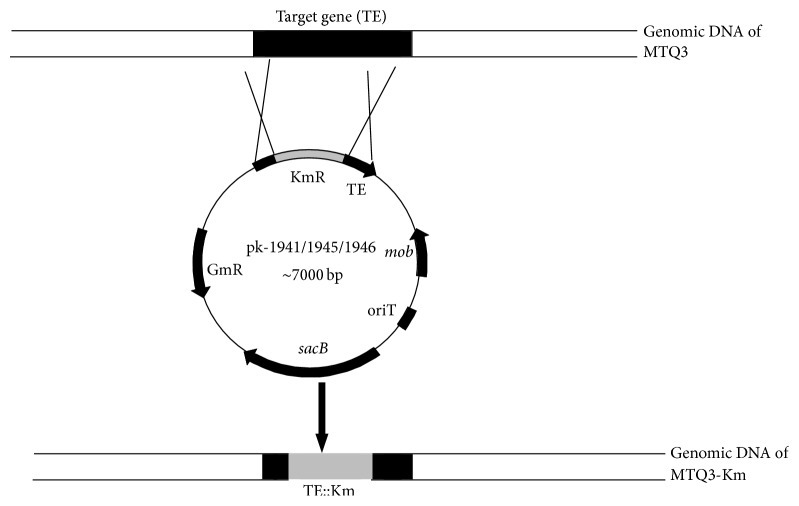
Knockout target genes with the Km cassette. The target genes orf-1941, orf-1945, and orf-1946 were amplified from the genomic DNA of MTQ3, after a series of enzyme digestions and ligation to form the recombinant plasmids pK-1941, pK-1945, and pK-1946. Triparental mating was used to generate homologous recombinant strains.

To reveal the function of the NRPS genes (Figure 4(a)), gene knockouts were performed. The amplified target fragments of three NRPSs were ligated into the pGEM-T easy vector between sites* Spe* I and* Xho* I, which were then religated to the suicide plasmid pJQ200SK using the same restriction endonucleases for cutting. The resulting suicide plasmids were then linearized by* Pst* I. Km fragments were cloned from the plasmid pUC4k by* Pst* I digestion and then ligated into the above linearized suicide plasmids. Finally, the resulting three plasmids pk-1941, pk-1945, and pk-1946 were transformed into* E. coli* DH5*α* [30]. With the help of plasmid pRK2013, triparental mating [31] was used to generate the recombinant strains MTQ3-Δ1941, MTQ3-Δ1945, and MTQ3-Δ1946 (Figure 5). The three mutants were selected by 5% sucrose plus LB plates with kanamycin and rifampicin and further confirmed by PCR using primers for Km and sequencing.

### 3.4. Qualitative and Quantitative Analyses of Siderophores

**Figure 6:**
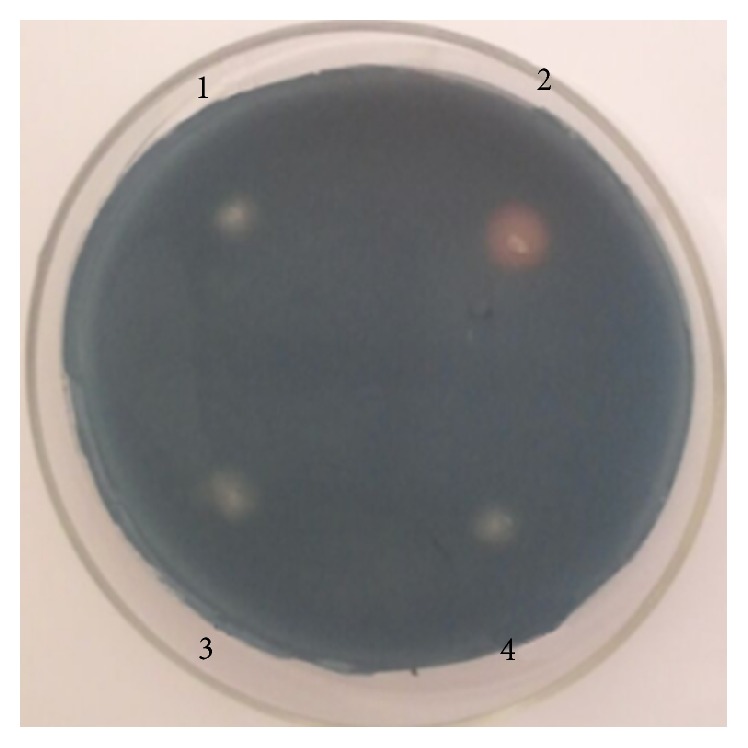
Qualitative analysis of siderophores on the CAS-agar plate. The bacterial lawn was inoculated on the CAS-agar plate for cultivation at 37°C for 2-3 days, followed by monitoring for a color ring. (1) MTQ_3_-Δ1941, (2) MTQ3, (3) MTQ_3_-Δ1945, and (4) MTQ_3_-Δ1946.

On the CAS-agar plates, we observed an orange halo around the colony of MTQ3 after 2-3 days of incubation, but no orange ring around the mutants (Figure 6). The presence of the orange ring suggested that MTQ3 can produce siderophores to chelate iron in the medium, thus resulting in a color change of the medium surrounding the colony. The mutants lost the ability to produce siderophores.

The quantitative measurements of siderophores (Table 4) indicated that the siderophore units of the wild-type strain MTQ3 were 47.8%. However, production levels by MTQ3-Δ1941, MTQ3-Δ1945, and MTQ3-Δ1946 were just less than 5%, which were regarded as negative results. In addition, we observed a color change from blue to orange when mixing the MTQ3 culture with the CAS assay solution, but this phenomenon was not observed for MTQ3-Δ1941, MTQ3-Δ1945, and MTQ3-Δ1946. This further demonstrated that NRPS mutants MTQ3-Δ1941, MTQ3-Δ1945, and MTQ3-Δ1946 failed to produce siderophores.

## 4. Discussion

In this study,* D. tsuruhatensis* MTQ3 was examined as a PGPR. The phylogenetic analyses showed that MTQ3 has high homology with* D. tsuruhatensis*. General features and a comparative genomic analysis with* Delftia* Cs1-4, SPH-1, and strain 391 suggested that* D. tsuruhatensis* MTQ3 shows some similarities with respect to COG categories, but the proportions are somehow different. MTQ3 contains a larger proportion of genes involved in carbohydrate transport and metabolism, which indicates its better potential for carbohydrate utilization. For secondary metabolite prediction using anti-SMASH, the gene clusters of NRPS and bacteriocin synthesis genes are not identical in the* Delftia* genomes. The different features of the four genomes may be explained by adaptations of strains to their specific environments.

As PGPR, MTQ3 exhibits the potential to produce siderophores. To determine the genes involved in siderophore production, three NRPS genes orf-1941, orf-1945, and orf-1946 of MTQ3 were figured out to test the function. They belong to one gene cluster, the genes of which show 85% similarity to the nonribosomal peptide metallophore, delftibactin [32, 33]. Compared with the wild-type strain MTQ3, three obtained mutants lost their ability to produce siderophores. These results indicated that siderophores could be biosynthesized by the NRPS modular multienzymes in MTQ3. Siderophores could improve the absorption of iron by plants, therefore promoting growth [4]. The ability of siderophores produced by bacteria to repress phytopathogens could be of significant importance in agriculture. This may explain why* D. tsuruhatensis* MTQ3 could stimulate plant growth. To the best of our knowledge, this is the first study to verify the gene cluster for siderophore production in* D. tsuruhatensis*. Meanwhile, the PGPR of MTQ3 may have important applications in agriculture.

## Figures and Tables

**Table 1:** Bacterial strains and plasmids used in this work.

Strain/plasmid	Relevant characteristics	Source
*D. tsuruhatensis *		
MTQ_3_	Wild-type strain, Rif^r^, Km^s^	[11]
MTQ_3_-Δ1941	*D. tsuruhatensis *MTQ_3-_Δ1941, Rif^r^, Km^r^	This work
MTQ_3_-Δ1945	*D. tsuruhatensis *MTQ_3-_Δ1945, Rif^r^, Km^r^	This work
MTQ_3_-Δ1946	*D. tsuruhatensis *MTQ_3-_Δ1946, Rif^r^, Km^r^	This work
*E. coli *		
DH5*α*	Host of recombinant plasmids	TransGen
*Plasmids*		
pJQ200SK	Suicide plasmid carrying *sacB*, Gm^r^, selecting double crossover MTQ_3_	[12]
pk-1941	pJQ200SK carrying Km and the fragment of orf-1941, Gm^r^, Km^r^	This work
pk-1945	pJQ200SK carrying Km and the fragment of orf-1945, Gm^r^, Km^r^	This work
pk-1946	pJQ200SK carrying Km and the fragment of orf-1946, Gm^r^, Km^r^	This work
pUC4K	Carrying Km cassette (*Pst *I), Km^r^	[13]
pRK2013	Helper plasmid used in triparental mating, Km^r^, Rif^s^	[14]
pGEM-T easy	TA cloning vector, Amp^r^	Promega

**Table 2:** Oligonucleotides used in this study.

Primers	Sequence (5′-3′)	Purpose
J1941F	GGACTAGTCTTTGGCGTGCCCGATGT (*Spe *I)	Cloning the fragment of orf-1941
J1941R	CCGCTCGAGTCGTTGGCGATGAGGTTGC (*Xho *I)	
J1945F	GGACTAGTTCCCTGAACGATCTCGATTCCC (*Spe *I)	Cloning the fragment of orf-1945
J1945R	CCGCTCGAGCATAGGTGCCACCGGCCTTG (*Xho *I)	
J1946F	GGACTAGTGCTTCCGCTGATCGACCTCA (*Spe *I)	Cloning the fragment of orf-1946
J1946R	CCGCTCGAGCGCCTTCTTCATCCTGCTCC (*Xho *I)	
KF1	CCCATCATCCAGCCAGAAAGTG	Cloning the fragment of Kna
KR1	ATAATGTCGGGCAATCAGGTGC	
27F	AGA GTT TGA TCC TGG CTC AG	Cloning the fragment of 16 srDNA
1492R	TAC GGC TAC CTT GTT ACG ACTT	

**Table 3:** General features of *D. tsuruhatensis* MTQ3 and other related genomes.

	*D. tsuruhatensis*	*Delftia *sp.	*D. acidovorans*	*D. tsuruhatensis*
	MTQ3	Cs1-4	SPH-1	391
Genome size	5,737,182	6,685,842	6,767,514	6,732,149
CDS number	4976	5861	6040	4103
G + C percentage	66.90%	66.71%	66.47%	66.30%
RNA number	92	98	98	76

**Table 4:** Quantification of siderophores.

Strain	Siderophore units (%)
MTQ3	47.8 ± 0.87
MTQ3-Δ1941	1.69 ± 0.64
MTQ3-Δ1945	0.72 ± 0.48
MTQ3-Δ1946	3.86 ± 0.48
